# Performance of molecular methods for the detection of *Salmonella *in human stool specimens

**DOI:** 10.12688/wellcomeopenres.16305.2

**Published:** 2021-05-04

**Authors:** Angeziwa Chunga Chirambo, Tonney S. Nyirenda, Ndaru Jambo, Chisomo Msefula, Arox Kamng'ona, Sandra Molina, Wilson L. Mandala, Robert S. Heyderman, Miren Iturizza-Gomara, Marc Y.R. Henrion, Melita A. Gordon

**Affiliations:** 1 Malawi-Liverpool-Wellcome (MLW) Programme, Blantyre, +265, Malawi; 2 Pathology Department, College of Medicine, Malawi, Blantyre, Malawi; 3 Institute of Infection, Veterinary and Ecological Sciences, University of Liverpool, Liverpool, L69 3BX, UK; 4 Biomedical Sciences Department, College of Medicine, Malawi, Blantyre, Malawi; 5 London School of Hygiene and Tropical Medicine, London, WC1E 7HT, UK; 6 Malawi University of Science and Technology, Thyolo, +265, Malawi; 7 Research Department of Infection, Division of Infection and Immunity, University College London, London, WC1E 6EJ, UK; 8 Department of Clinical Sciences, Liverpool School of Tropical Medicine, Liverpool, L3 5QA, UK

**Keywords:** Salmonella Typhi, nontyphoidal Salmonella, bacteremia, gastrointestinal tract, diagnostics, stool culture, polymerase chain reaction

## Abstract

**Background:** The relationship between asymptomatic *Salmonella* exposure within the gastrointestinal tract and *Salmonella* bacteraemia is poorly understood, in part due to the low sensitivity of stool culture and the lack of validated molecular diagnostic tests for the detection of *Salmonella* in the stool. The study aimed to determine a reliable molecular diagnostic test for *Salmonella* in stool specimens.

**Methods:** We optimised an in-house monoplex real-time polymerase chain reaction (PCR) for the detection of *Salmonella* *ttr* and *InvA* genes in stool by including a selenite broth pre-culture step for *Salmonella* before DNA extraction and validated their specificity against other local common pathogens. Then we assessed their performance against a well-validated multiplex PCR targeting the same *ttr* and *InvA* genes and against stool culture using clinical stool specimens collected from a cohort of 50 asymptomatic healthy Malawian children that were sampled at 1-month intervals over 12 months. We employed a latent Markov model to estimate the specificities and sensitivities of PCR methods.

**Results**: *Ttr* and *InvA* primers were both able to detect all the different *Salmonella* serovars tested and had superior limits of detection when DNA was extracted after selenite pre-culture. T *tr* sensitivity and specificity for monoplex-PCR were (99.53%, 95.46%) and for multiplex-PCR (90.30%, 99.30%) respectively. *InvA* specificity and specificity for using monoplex-PCR was (95.06%, 90.31%) and multiplex-PCRs (89.41%, 98.00%) respectively. Sensitivity and specificity for standard stool culture were 62.88% and 99.99%, respectively. Culture showed the highest PPV (99.73%), and monoplex- *ttr* had the highest NPV (99.67%).

**Conclusion:** Test methods demonstrated high concordance, although stool culture and monoplexed *ttr* primers had superior specificity and sensitivity, respectively. The use of selenite pre-enrichment step increased *Salmonella* detection rate. Taken together, molecular detection methods used here could be used to reveal the true extent of both asymptomatic and symptomatic *Salmonella* exposure events.

## Introduction

*Salmonellae* cause a huge global burden of morbidity and mortality. They are globally estimated to be responsible for 300,000 deaths ^1– 4^. *Salmonella enterica* serovars Typhi and Paratyphi A are the predominant cause of invasive *Salmonella* infections in south and southeast Asia and cause between 129,000 to 223,000 global deaths per year ^1, 3, 5^. In contrast, non-Typhoidal *Salmonella* (NTS) serovars, principally *S. Typhimurium* and *S. Enteritidis*, are a common cause of invasive disease in sub-Saharan Africa (SSA) ^4, 6^. In 2017, NTS caused an estimated 535,000 cases, with SSA having the highest incidence ^7^. Risk factors for invasive NTS (iNTS) disease include young age, recent malaria, and advanced HIV disease. Case fatality rates for iNTS in young children, people infected with HIV, and living in the SSA region were estimated at 13.5%, 41.8%, and 15.8%, respectively ^4^. This is in marked contrast to the presentation of *Salmonella* disease in high-income countries, where NTS typically cause a self-limiting diarrhoeal disease in healthy individuals, while bloodstream or focal infections are rare and mainly occur in individuals with specific risk factors such as diabetes, neoplastic and autoimmune disease, or immunosuppressive therapy ^8^. However, it is notable in both settings that invasive NTS disease in adults and children is not always associated with diarrhoea ^9^.

We previously described in under-five-year-old children the sequential development of cellular and humoral immunity against the *Salmonella* serovars causing iNTS disease, and that acquisition of this immunity is associated with decreasing incidence of disease ^10, 11^, suggesting that this immunity is protective. Previous studies have reported that healthy young children experience transient asymptomatic episodes of gastrointestinal infection with non-typhoidal *Salmonella* ^12, 13^, and we, therefore, hypothesise that episodes of asymptomatic *Salmonella* exposure in the healthy gastrointestinal tract during early childhood may facilitate the development of protective immunity. Balanced against this beneficial effect of exposure, diarrhoeal disease results from enteric *Salmonella* exposure, and invasive NTS disease also follows episodes of asymptomatic gastrointestinal exposure in susceptible children, including those with malaria or malnutrition, or immunocompromised individuals.

Elucidating the relationship between *Salmonella* exposure events within the gastrointestinal tract and resultant Salmonella immunity or Salmonella disease is critical for understanding iNTS disease pathogenesis. Lack of affordable and rapid diagnostic tools for the detection of bloodstream and intestinal *Salmonella* disease hampers our understanding of *Salmonella* disease epidemiology and pathogenesis. Blood culture is considered the gold standard diagnostic test for *Salmonella* bacteremia and is highly specific but has a number of drawbacks; poor turnaround time of between 2 to 7 days, and low sensitivity of about 20% – 30% for samples collected 7 days post-infection ^14– 16^. Molecular detection of *Salmonella* in blood also has limited apparent sensitivity, and various assays are in development ^13, 17^.

Stool culture is similarly considered the gold standard test for the detection of *Salmonella* in the intestinal tract. However, stool culture, even for diarrhoeal disease when the bacterial load is likely to be high, has poor sensitivity (<50%), and is labour and time-consuming ^18^. Real-time PCR has a short turnaround time and is potentially highly sensitive compared to standard culture, and has the capacity for automation and testing for multiple targets ^19^. However, stool PCR test performance is hindered by PCR inhibitors and a large number of genetically closely related enteric bacteria. These pose a challenge in generating highly specific and sensitive primers for real-time PCR (qPCR) for *Salmonella*. Furthermore, a lower infective load of *Salmonella* colonisation during asymptomatic infection may further limit detection by PCR.

With this background, we validated an in-house monoplex qPCR method for the detection of *Salmonella* in stool specimens and compared them with a validated multiplex-based qPCR and standard stool culture. Both qPCR assays used primers and probes based on the *Salmonella* tetrathionate respiration gene ( *ttr*) and the *Salmonella* invasion gene A ( *InvA*). Stool specimens were collected from healthy, mainly asymptomatic healthy Malawian children aged 6–18 months. Assessing a diagnostic test's performance is challenging when the existing “gold standard” test being used has known low sensitivity or specificity. Statistical methods, such as the Latent Markov model, are used to assess diagnostic tests' performance without assigning a gold standard test. Since the current reference standard is known to lack sensitivity, we employed a latent Markov model to estimate the specificities and sensitivities of PCR methods without assigning a gold standard.

## Methods

### Description of study participants and specimens

Stool specimens collected from a longitudinal cohort of children aged 6 – 18 months recruited from Zingwangwa Health Centre (ZHC) in Blantyre, Malawi, were used to compare the performance of molecular and standard culture for detection of *Salmonella* in stool. The primary study started recruitment in August 2013, and follow-up was concluded in December 2014 ^17^. Group sensitisation of the study by well-trained study nurses was done to parents or guardians of six-month-old children attending a vaccination clinic at ZHC. Individual sensitisation was also done to parents or guardian that were interested in joining the study. Children who met the inclusion criteria of being healthy were recruited into the study after obtaining consent. Children born preterm (less than 38 weeks gestation), HIV positive or HIV exposed, and those with fever >38°C or any acute illness were excluded from the study ^20^.

Stool samples were collected monthly until they were aged 18 months. Stool specimens were collected in sterile and clean containers and transported to the laboratory on the same day. From 60 children recruited at 6 months of age, 10 children withdrew from the study, and 600 stool specimens were collected and tested by culture on the day of sample collection at the College of Medicine and Malawi Liverpool Wellcome Laboratory. Molecular tests were done on frozen samples that were available at the time the tests were done.

### *Salmonella* stool culture

A matchstick head-size sample of stool was inoculated in 10 ml of selenite F broth (Oxoid, UK, catalog number: 2300631) and aerobically incubated overnight at 37 °C for 18–24 hours. The top layer (1 ml) of an overnight culture was spun at 20,000 g for 5 minutes. This is a method that has been developed in our laboratory. A 1 ul loop was used to subculture *Salmonella* from the pellet by spreading on Xylose Lysine Deoxycholate (XLD) agar (Oxoid, UK, catalog number: 2547703) to achieve single colonies. Careful plate spreading prevents overcrowding of colonies. A single colony was picked and cultured on sheep blood agar. An aliquot of the selenite broth was also frozen for molecular detection. A single colony of presumptive *Salmonella* was cultured onto sheep blood agar (Oxoid, UK, catalog number: 2910831) and MacConkey agar plates (Oxoid, UK, catalog number: 2529552) and incubated aerobically at 37°C for 18–24 hours. *Salmonella colonies* were then distinguished from other enteric bacteria (i.e., *Citrobacter* and *Serratia*) using triple sugar iron agar (Oxoid, UK, catalog number: 1882283) and Urea agar (Oxoid, UK, catalog number: 1779617) biochemical tests. Further *Salmonella* identification was determined using API® 10S (bioMérieux, France, catalog number: 1007181060) according to the manufacturer’s instructions.

### Monoplex-qPCR *ttr* and *InvA* assay

***Validation of the monoplex- qPCR ttr and InvA assay.*** For the monoplex-qPCR, the *ttr* primers and probe were designed and validated by Federal Institute for Risk Assessment, Berlin, Germany, according to the published DNA sequence of the *S. enterica* serotype Typhimurium *ttr* locus for *Salmonella* detection (GenBank accession no. AF282268). The use of *ttr* was based on 21. In this study, the ttr gene's specificity was assessed using 110 *Salmonella* strains representing 31 serotypes, and it demonstrated 100% specificity. The *InvA* gene has been widely studied and used as a pan *Salmonella* marker. In 22, the specificity of the *InvA* gene was assessed using 242 *Salmonella* strains representing 43 serotypes. It also demonstrated 100% specificity. *InvA* has shown 100% specificity in several other studies. ** Both primers required optimisation for use in stool specimens. The DNA sequences of all the primers and probes used in this study are listed in Table 1.

**Table 1.** List of primers and probes sequences used in this study. Primers and probes sequences used in this study include in-house designed *InvA*, *ttr* previously validated for *Salmonella* detection in food, and TAC- *InvA* and TAC- *ttr* used on a well-validated TAC assay as pan *Salmonella* primers.

	Primer name	Primer direction	Primer code/Probe description
1	*INVA*	Forward	5’-AGCGTACTGGAAAGGGAAAG-3’
2	*INVA*	Reverse	5’-CACCGAAATACCGCCAATAAAG-3’
3	*INVA*	Probe	FAM-TTACGGTTCCTTTGACGGTGCGAT-BHQ1
4	*ttr*	Forward	5’-CTCACCAGGAGATTACAACATGG-3’
5	*ttr*	Reverse	5’-AGCTCAGACCAAAAGTGACCATC-3’
6	*ttr*	Probe	FAM-CACCGACGGCGAGACCGACTTT-BHQ1
7	*InvA*-TAC	Forward	5’-GGCAATTCGTTATTGGCGATA-3’
8	*InvA*-TAC	Reverse	5’-CACGGTGACAATAGAGAAGACAACA-3’
9	*InvA*-TAC	Probe	FAM-CCTGGCGGTGGGTT-MGB
10	*ttr*-TAC	Forward	5’-CTCACCAGGAGATTACAACATGG-3’
11	*ttr*-TAC	Reverse	5’-AGCTCAGACCAAAAGTGACCATC-3’
12	*ttr*-TAC	Probe	FAM-CACCGACGGCGAGACCGACTTT-MGB

### Specificity of *ttr* and *InvA* primer/probe set for *Salmonella* compared to other local pathogens

To determine the prevalence of ttr and *InvA* in the genomes of *Salmonella* serotypes, NCBI nucleotide blast was conducted (10 February 2021). Ttr and *InvA* nucleotide sequences were used as the query sequence against *Salmonella* enterica subspecies enterica (taxid:59201) genomes with a maximum target sequence set at 1000. Both primer sequences demonstrated 100% identity for most commonly isolated *Salmonella* serotypes (supplementary material). To determine the specificity of the primers *in vitro,* 9 different locally isolated and whole genome sequenced *Salmonella* strains and 26 pure isolates of non- *Salmonella* bacterial strains locally isolated from blood culture were tested using *ttr* and *InvA* primer/probe sets ( Table 2). These 9 *Salmonella* strains represented all known *Salmonella* serotypes in the MLW isolate archive. The non- *Salmonella* strains were chosen because they are genetically closely related to *Salmonella* or because their growing conditions are similar to *Salmonella*. These strains were collected from MLW bacterial blood culture repository. Overnight cultures of the frozen samples were made on SBA or LB agar. One colony was then cultured in liquid media. After reaching stationary growth phase, a known and matched concentration of about 10 ^6^ CFU was used for DNA extraction using QIAamp Fast DNA Stool Mini Kit (QIAGEN, Netherlands, catalog number: 51604) but without the bead beating step. Miles and Misra technique was used for bacteria quantification.

**Table 2.** Bacterial organisms tested for the specificity of *ttr* and *InvA* primer/probe sets. Bacterial organisms used in this study to test for the specificity of *ttr* and *InvA* primer/ probe sets. Nine *Salmonella* and 26 non- *Salmonella* isolates previously isolated at MLW laboratory were retrieved and tested either as direct or selenite sub-cultured isolates.

Bacteria isolates	Number tested	Direct		Selenite sub- cultured	
		*ttr* Positive	*InvA* Positive	*ttr* Positive	*InvA* Positive
*Morganella morgana*	1	0	0	0	0
*Streptococcus pneumonia*	1	0	0	0	0
*Staphylococcus aureus*	1	0	0	0	0
*Citrobacter*	1	0	0	0	0
*Klebsiella*	1	0	0	0	0
*Enterobacter*	1	0	0	0	0
*Acinetobacter*	1	0	0	0	0
*Enterobacter intermedius*	1	0	0	0	0
*Enterococcus feacium*	1	0	0	0	0
*E. coli*	17	0	0	0	0
*S. Typhi*	1	1	1	1	1
*S. Typhimurium*	1	1	1	1	1
*S. Enteritidis*	1	1	1	1	1
*S. Braenderup*	1	1	1	1	1
*S. Virchow*	1	1	1	1	1
*S. Bonn/Fann*	1	1	1	1	1
*S. Oesterbro/Zanzibar*	1	1	1	1	1
*S. Heidelberg*	1	1	1	1	1
*S. Dublin*	1	1	1	1	1

**Limits of detection in different conditions** A well-characterised invasive *S.* Typhimurium ST313 strain (D23580), isolated from an HIV-negative child in Malawi, and representative of our commonest invasive bloodstream infections, was used as a reference strain for determining limits of detection in varying kinds of sample ^23, 24^. Three types of Salmonella samples were prepared for comparison using RT-PCR; 1) pure *Salmonella* isolates picked from a blood agar plate, 2) *Salmonella* cultured in selenite broth, and 3) *Salmonella* spiked into stool. *Salmonella* stool spiking was done to determine the inhibitory effect that stool may have on the assay, affecting the limit of detection. For this, a stool sample was collected from a healthy individual and confirmed as *Salmonella* negative by culture. The stool sample was thereafter diluted with PBS (50% w/v) and then spiked with *S*. Typhimurium, D23580, at varying doses of viable bacteria *.* The viable dose of *Salmonella* was adjusted across a range from 10 ^0^–10 ^6^ CFU/ml and quantified using Miles and Misra technique. DNA was extracted for RT-PCR, as above. All experiments were repeated three times on different days by the same operator.

***Detection of Salmonella in clinical samples using monoplex-qPCR ttr and InvA assay.*** The primer/probe sets were then used to detect *Salmonella* in clinical stool samples collected from the longitudinal cohort study of healthy asymptomatic children. For the monoplex qPCR, approximately 200μl top layer of frozen Selenite F broth overnight stool culture, or 200 mg of stool, was suspended in 500 μl of PBS. DNA was extracted using QIAamp Fast DNA Stool Mini Kit (QIAGEN, Netherlands, catalog number: 51604) according to the manufacturer’s instructions, with an added bead-beating step. Eluted DNA was stored at –20°C.

A previously-optimised in-house PCR protocol was used ^17^. Briefly, the master mix for RT-PCR was prepared using pre-defined quantities. A total of 20μl master-mix for each sample was comprised of the following: 12.5μl Platinum *®* Quantitative PCR Super Mix-UDG (Life Technologies, USA, Catalog number: 11730025), 0.10μl specific forward primer, 0.10 specific reverse primer, 0.10 specific probe (all primers and probes at 200nM), 0.05μl ROX reference dye (Life Technologies, USA, Catalog number: 12223012) at 50nM final concentration, and 7.15μl nuclease-free water. This mixture was transferred to 96-well plate PCR wells. 5μl of test DNA, positive controls DNA (DNA from D23580), technical extraction negative control, and assay negative control (UV treated water) were added in triplicates to appropriate wells containing 20ul of master-mix. The qPCR was run for 40 cycles using Applied Biosystems® 7500 Real-Time PCR Systems (Life Technologies, USA). The following cycling conditions were used; initial denaturation at 95°C for 1 minute, denaturation at 95°C for 15 seconds, annealing/extension at 60°C for 30 seconds, final extension: 12°C. The threshold was set in the lag phase. An assay was considered to have passed when the positive controls were positive, and both the technical extraction negative and assay negative controls were negative. Test sample cycle threshold (Ct) values were evaluated after subtracting the baseline value. Samples with cycle threshold (Ct) values of less than or equal to 35 were considered positive.

### Detection of *Salmonella* using multiplex qPCR assay

As a comparator, we used a well-validated TAC assay on DNA extracted from stool samples, according to the manufacturer’s protocol. The customised Taqman Array Card assay developed and validated at the University of Virginia was used. The performance of the TAC method has been previously described and has now been widely used ^12, 25– 27^. It is used to detect multiple enteric pathogens, including bacteria, viruses, protozoa, and helminths. Targets included on the TAC card for pan *Salmonella* detection are *InvA* and *ttr* ^28^. Phocine Herpesvirus (PHhv) and MS2 targets are included as internal positive controls.

To extract total nucleic acid (TNA) from the clinical samples for TAC assay, we used QIAamp Fast DNA Stool Mini Kit (QIAGEN, Netherlands, catalog number: 51604) - the same DNA extraction kit and protocol that were used to extract whole-stool DNA for the monoplex qPCR assay, with the addition of internal extraction positive controls. For TNA extraction, each sample was extracted together with internal positive controls, Phocine Herpesvirus (PHhv), and MS2. PHhv and MS2 were added to the inhibitX buffer before being added to each sample, as previously described ^28^. An assay was considered to have passed when both MS2 and PhHv internal positive (amplification crossing the threshold) and negative controls (no amplification crossing the threshold line) passed and when the sample had a sigmoid curve that crossed the threshold line. A sample was classified as pathogen positive at a Ct value of <35. Only results for *Salmonella* are reported here.

### Statistical analysis

Data were recorded and analysed in MS Excel (version 16.14.1 (18061302)). Sensitivities and specificities of the different PCR methods were estimated using a latent Markov model (LMM) ^29^. We have previously described the LMM and various extensions that we considered for modeling longitudinal diagnostic test data ^30^. We implemented the LMM within a Bayesian framework using R (version v3.5.1) and JAGS (version 4.3.0) via the rjags (version 4.6) R package ^31^. LMMs have been extensively used for discrete-time longitudinal data in the absence of a gold standard diagnostic procedure ^32, 33^. LMMs consists of a process model for a latent condition (in our case, the unobserved true infection status) evolving over time and a measurement model for the observed outputs (in our case, the results from the 5 diagnostic test methods) conditional on the latent state. We considered several LMMs, with and without mixed effects and with either time-homogeneous or time-heterogeneous transition matrices ^30^. Convergence and identifiability of the LMM were checked by inspecting trace plots and computing Gelman-Rubin potential scale reduction factors ^34^. The more complex models exhibited poor mixing or convergence of MCMC chains (most likely due to the sparse number of positive samples). As a result, the LMM we used for this dataset is a basic LMM with no random effects and a time-homogeneous transition matrix. To report positive predictive values (PPV) and negative predictive values (NPV), we calculated an estimate of the infection prevalence. For the Bayesian LMM, we report maximum *a posteriori* (MAP) parameter estimates together with 95% credible intervals (Crl), specifically the highest posterior density intervals (HDI) with 95% coverage. All other analyses report (frequentist) parameter estimates and corresponding 95% confidence estimates (CI).

### Ethical considerations

Ethical approval for this work was granted by the University of Malawi, College of Medicine Research Ethics Committee (P.01/13/1327). Written informed consent was obtained from the parent or guardian of each participating child.

## Results

### *Ttr* and *InvA* primers for Salmonella do not cross-react with closely related enteric micro-organisms

We first validated the *ttr* and *InvA* primers that were used in the monoplex-qPCR assay by assessing the sensitivity and specificity of the primers for Salmonella, using a standardised number of 10 ^0^–10 ^6^ CFU/ml of 9 different locally-relevant *Salmonella* strains and 26 non- *Salmonella* bacterial strains as indicated in Table 2. We included 17 strains of *E*. *coli* because of the close genomic relatedness of *Salmonella* and *E. coli*. Bacterial isolates enriched in Selenite F broth (referred here as selenite sub-cultured) or not (referred here as direct culture) were used in this evaluation. We found that *ttr* and *InvA* assays both achieved 100% sensitivity and specificity either as direct isolates or selenite sub-cultured isolates. Table 2 demonstrates that all *Salmonella* strains tested positive with both monoplexed primer pairs, and all other bacterial strains were negative, confirming a lack of cross-reactivity. Additionally, the NCBI nucleotide blast demonstrated that the genomic prevalence of *ttr* and *InvA* genes is high. Both primer sequences demonstrated 100% identity for most commonly isolated Salmonella serotypes.

### Selenite broth culture enhances the detection of Salmonella in stool using either *ttr* or *InvA* primers

The limits of detection (LOD) of qPCR for *Salmonella* were then determined using *S.* Typhimurium strain D23580 serially diluted and tested as direct isolates, selenite broth cultured samples, or isolates spiked into a culture-negative stool specimen. We found that limits of detection for *ttr* were 1, 10, and 100 CFU/ml, and for *InvA* were 1, 100, and 100 CFU/ml for selenite sub-cultured broth, direct isolates, and stool-spiked isolates, respectively, with 98.5% qPCR efficiency for *ttr* and 97.2% qPCR efficiency for *InvA*. No statistically significant difference was observed in the LOD when *ttr* was compared with *InvA* in either direct isolates (p = 0.3212), selenite sub-cultured samples (P = 0.2534), or *salmonella* spiked stool samples (P = 0.2361). Importantly, we found that the *ttr* assay was significantly different when direct isolates (LOD = 10 CFU/ml) were compared with selenite sub-cultured samples (LOD = 1 CFU/ml) (p<0.0001), and when selenite sub-cultured isolates were compared to *Salmonella* spiked stool ( *p* <0.0001), and there was no significant difference when direct isolates were compared to *Salmonella* spiked stool ( *p*=0.2965).

Similarly, we found that detection in *InvA* qPCR assay direct isolates was significantly different compared to selenite broth cultures isolates ( *p* < 0.0001), and selenite subculture isolates were also significantly different from *Salmonella* spiked stool ( *p* < 0.0001). In contrast, there was no significant difference between direct isolates compared to *Salmonella* spiked stool samples ( *p* = 0.2862). In summary, we found that selenite broth overnight liquid culture of stool samples enhanced the molecular detection of *Salmonella* using either *ttr* or *InvA* primers, even if culture of the broth remained negative.

### *Ttr* and *InvA* primers had both high specificity and sensitivity rates, while stool culture had high specificity but low sensitivity

The samples from healthy children were used to determine the performance of stool culture, monoplex *ttr*, monoplex *InvA*, multiplex TAC *ttr*, and multiplex TAC *InvA*. Standard stool culture was performed on a total of 600 specimens at different time points. Molecular tests were used to detect *Salmonella* in the available 421 stool DNA specimens. We detected *Salmonella* in 23, 40, 29, 56, and 47 of 421 stool specimens, using standard stool culture, *ttr*, *InvA*, TAC- *ttr*, and TAC- *InvA* respectively. Of the 23 *Salmonella* stool culture-positive samples, 21 samples were also positive with either one or more molecular tests, while 2 were molecular tests negative.

**Figure 1.**
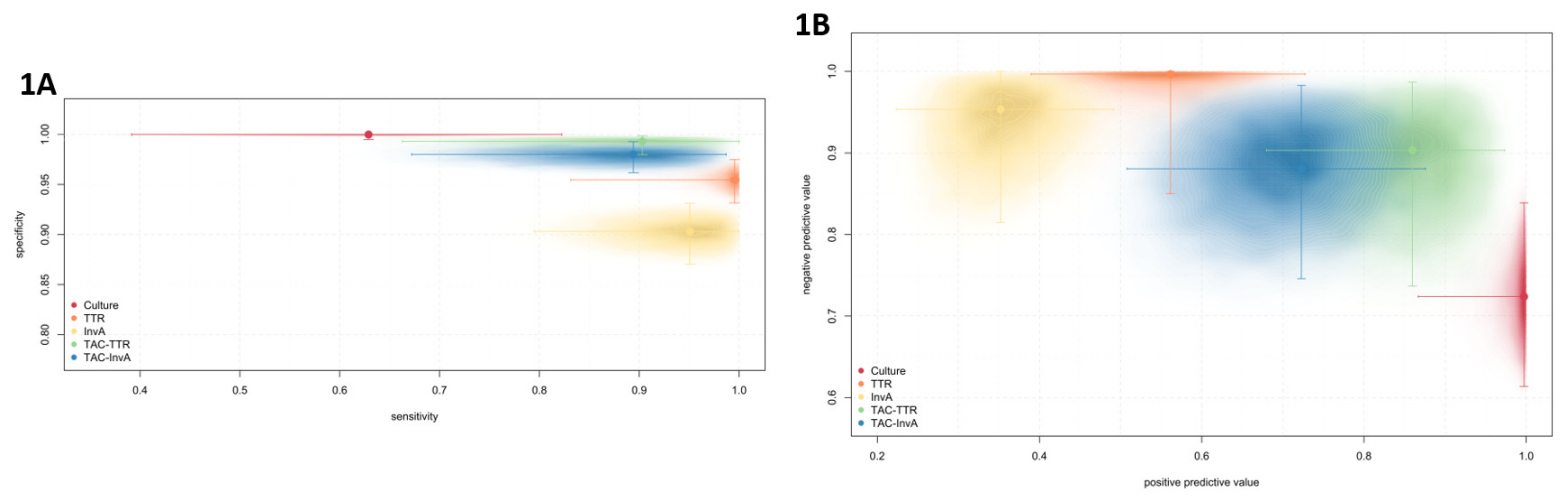
Maximum a posteriori probability estimates of the specificities and sensitivities (Figure **1A**), positive and negative predictive values (Figure **1B**) together with 95% highest density credible intervals (segments) and posterior density estimates (contours) for stool culture, *ttr*, *InvA*, TAC- *ttr,* and TAC. Big dots and error bars represent the median values and 25 and 75 percentile.

Based on a time-homogeneous LMM without random effects ( Table 3 and Figure 1A), we report the specificities and sensitivities of the detection methods with their 95% credible intervals (Bayesian confidence intervals). The observed specificity rates from highest to lowest were for stool culture (99.99%), TAC- *ttr* (99.30%), TAC- *InvA* (98.00%), monoplex *ttr* (95.46%), and monoplex *InvA* (90.31%), respectively. The observed sensitivity rates from highest to lowest were monoplex *ttr* (99.53%), monoplex *InvA* (95.06%), TAC- *ttr* (90.30%), TAC- *InvA* (89.41%,) and stool culture (62.88%) respectively ( Table 3 and Figure 1A). While stool culture achieved the highest specificity and monoplex *ttr* the highest sensitivity, monoplex *ttr* achieved arguably the best sensitivity-specificity trade-off: very high sensitivity (99.53%) at a relatively small drop in specificity (95.46) compared to stool culture.

**Table 3.** Probability estimates of the specificities and the sensitivities, PPV, and NPV of the diagnostic tests. Maximum a posterior probability estimates of the specificities and the sensitivities, PPV, and NPV of the diagnostic tests. Also reported are the 95% highest density credible intervals for each parameter.

	Sensitivity		Specificity		Positive predictive value		Negative predictive value	
	MAP	(95% Crl)	MAP	95% Crl	MAP	95% Crl	MAP	95% Crl
**Stool culture**	0.6288	(0.3916,0.8223)	0.9999	(0.9949,10000)	0.9973	(0.8668,10000)	0.7238	(0.6135,0.8389)
***ttr***	0.9953	(0.8315,1.0000)	0.9546	(0.9317,0.9749)	0.5615	(0.3897,07275)	0.9967	(0.8501,10000)
***InvA***	0.9506	(0.7950,10000)	0.9031	(0.8702,0.9311)	0.3521	(0.2233,0.4915)	0.9536	(0.8147,10000)
**TAC- *ttr***	0.903	(0.6628,10000)	0.993	(0.9797,0.9987)	0.8597	(0.6798,0.9736)	0.9033	(0.7367,0.9869)
**TAC- *InvA***	0.8941	(0.6721,0.9869)	0.98	(0.9618,0.9928)	0.7228	(0.5079,0.8757)	0.8807	(0.7459,0.9828)

### High negative and positive concordance for stool culture, monoplex *ttr*, monoplex *InvA*, Multiplex *ttr*, and multiplex *InvA*

**Figure 2.**
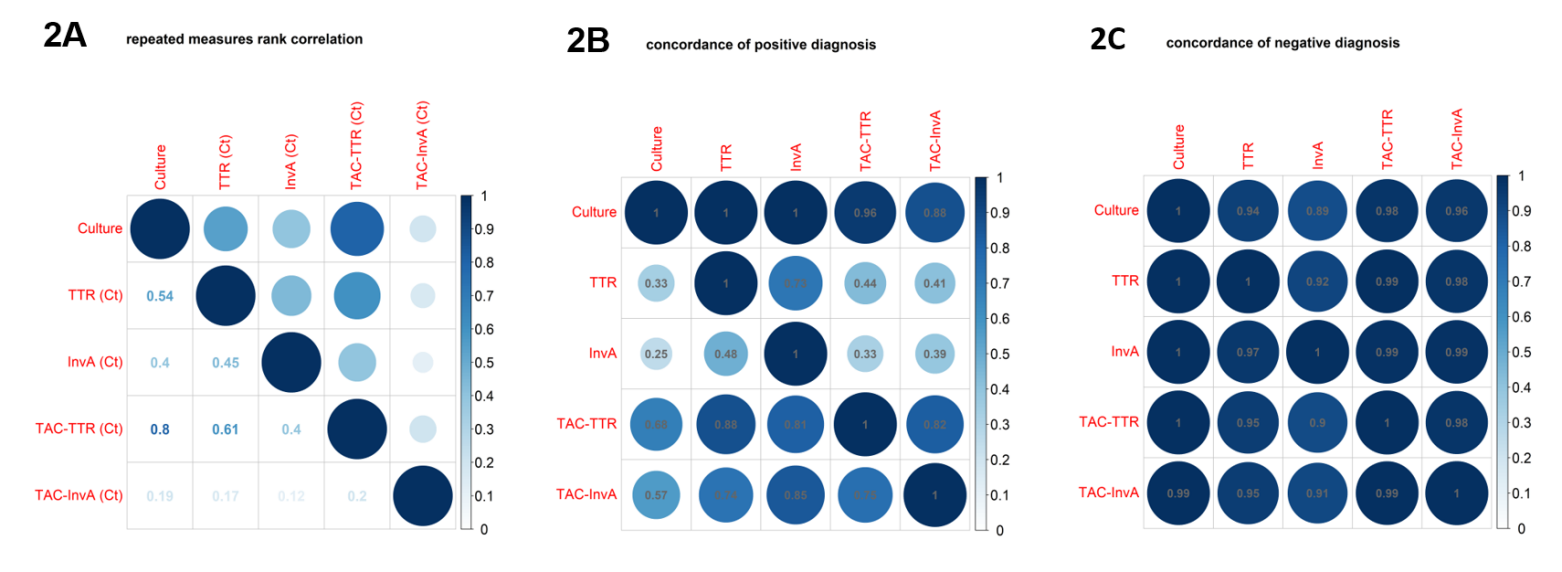
Correlation coefficients for the four molecular tests (using Ct values) and stool culture using positive or negative (Figure **2A**). Concordance coefficients for positive (Figure **2B**) and negative (Figure **2C**) diagnosis obtained using binary negative or positive outcomes for each test. For example, in Figure **2B**, the intersection of the row labeled ‘Culture’ and the column labeled ‘ *ttr*’ lists the proportion of positive test results for the Culture test that are also positive for the *ttr* test. Both the size and colour depth represent the magnitude of correlation.

Next, we explored correlations between stool culture, monoplex *ttr*, monoplex *InvA*, Multiplex *ttr*, and multiplex *InvA*. In this exploration, we considered all test results, whether positive or negative. To account for both censored observations and the longitudinal nature of the data, we calculated repeated measures of correlation coefficients ^35^ using the ranks of observations for each test (akin to a repeated-measures Spearman correlation coefficient) for measuring the correlation between the Ct values for the four molecular tests and point bi-serial correlation coefficients based on ranks for measuring correlations between standard stool culture and each of the qPCR tests ( Figure 2A). The correlation coefficients vary quite widely from 0.12 (monoplex *InvA* and TAC- *InvA*) to 0.8 (stool culture and TAC- *ttr*). Given that for truly negative samples, the Ct values are effectively randomly distributed near the threshold used to discriminate between positive and negative samples and that most samples were negative in most tests, the somewhat weak correlations we observe can be driven by the random Ct values for negative samples. For this reason, using only the binary negative / positive outcomes for each test, we computed positive ( Figure 2B) and negative ( Figure 2C) concordance: for example, in Figure 2B, the intersection of the row labeled ‘ *ttr,*’ and the column labeled ‘ *InvA*’ lists the proportion of positive test results for the *ttr* test that are also positive for the *InvA* test. Unexpectedly (given that most samples were negative), negative concordance ( Figure 2C) was very high, with the lowest negative concordance being 89%. Results for positive concordance ( Figure 2B) are also relatively high, though there is more variation, ranging from 25% (for positive *InvA* results confirmed by positive stool cultures) to 100% (positive stool cultures confirmed by positive monoplex *ttr* or positive monoplex *InvA*).

### Stool culture had high positive predictive value while molecular tests methods had high negative predictive values

To report PPV and NPV, for an estimate of prevalence, we use the model-estimated stationary (time-homogeneous model) probability of being infected (MAP 5.25%, 95% credible interval [3.27%, 8.14%]). From highest to lowest, the estimated PPVs were culture (99.73%), TAC- *ttr* (85.97%), TAC- *InvA* (72.28%), mono- *ttr* (56.15%), mono- *InvA* (35.21%). From highest to lowest, the estimated NPVs were mono- *ttr* (99.67%), mono- *InvA* (95.36%), TAC- *ttr* (90.33%), TAC- *InvA* (88.07%), and culture (72.38%) as indicated in Table 3 and Figure 1B. While stool culture has the highest PPV (99.73%) and mono- *ttr* the largest NPV (99.67%), a good trade-off between PPV and NPV is achieved by TAC- *ttr* (both high PPV, 85.97%, and high NPV, 90.33%). We note that these PPV and NPV estimates are for an asymptomatic population of children in urban Blantyre, Malawi, and are not directly generalisable to different contexts and populations where prevalence may be higher or lower.

## Discussion

The burden of asymptomatic gastrointestinal exposure to *Salmonella,* which could be linked to either the development of immunity or, conversely, to bloodstream infection, is not known due to lack of robust *Salmonella* detection methods for stool specimens. This study aimed to optimise detection methods and to validate and compare the performance of monoplex *ttr* and *InvA* qPCR assays ( *ttr* and *InvA*) against *ttr* and *InvA* qPCR assays on a validated multiplex qPCR platform (TAC- *ttr* and TAC- *InvA*), and compare all molecular methods to standard *Salmonella* stool culture. Validation of the monoplex *ttr* and *InvA* primers showed that the primers do not cross-react with other enteric pathogens, and LOD testing showed that selenite pre-culture promotes molecular detection, even when culture is negative. Stool culture demonstrated the highest specificity but low sensitivity than all the molecular tests. Stool culture, despite having low sensitivity, still remains important in *Salmonella* diagnosis. Culture allows for antimicrobial susceptibility testing and strain typing. *Ttr* detected on the monoplex platform demonstrated superior sensitivity to stool culture, *InvA*, TAC- *ttr*, and TAC- *InvA*. All the test methods, however, displayed high concordance to each other.

Several studies have developed *Salmonella* detection methods based on antigen detection or nucleic acid amplification ^16, 18, 36, 37^. Both monoplex and multiplex nucleic acid amplification-based detection methods have been developed ^38– 41^. Most of these have, however, focused on *Salmonella* detection in blood as opposed to stool specimens. Some multiplex qPCRs to specifically detect *Salmonella* and its serovars or for the detection of multiple enteric pathogens in stool specimen (including *Salmonella*) have recently been developed ^28, 42– 44^. The advantage of multiplex qPCR is that it is fast in determining the primary etiological agent in cases where multiple pathogens or different serovars cause the outcome, but it is expensive if one is interested in detecting only one particular pathogen ( Table 4). By contrast, the advantage of a monoplex test is that it is economical than the multiplex, faster than stool culture, even with the addition of the selenite enrichment step and allows for batch-processing of samples which increases the efficiency of the test method. In this study, the same primer/ probe sets were tested using both the monoplex and multiplex qPCR platforms. The monoplex qPCR maximised sensitivity, while the multiplex panel provided a balanced pay-off between sensitivity and specificity ( Table 4). The high sensitivities of the monoplex qPCR could be attributed to the use of selenite pre-cultured stool as opposed to extraction of DNA from neat stool samples, which is used in the multiplex qPCR. Selenite sub-cultured stool samples were not used on the multiplex platform because the manufacturer’s protocol was followed. Other studies have, however, also demonstrated superior performance of monoplex qPCR when compared with multiplex qPCR. The monoplex qPCR is therefore ideal for studies that are only interested in determining the presence or absence of *Salmonella* while capitalising on the sensitivity of the test, while multiplex qPCR will have an added advantage if a study wants to detect multiple pathogens while having a pay-off between sensitivity and specificity.

**Table 4.** Characteristics of *Salmonella* stool culture, Monoplex -qPCR *ttr,* Monoplex -qPCR *InvA,* Multiplex -qPCR *ttr and* Multiplex -qPCR *InvA*. A summary of the characteristics of the test methods. Specific values are indicated for sensitivity and specificity. Estimates were made for time to assay result, person-labour time requirement, cost of the test, specialist equipment cost by ranking them from Low to very high. Comparisons were also made for follow-up tests, antibiotic sensitivity and serotyping, and efficiency in batching.

	*Salmonella* stool culture	Monoplex -qPCR *ttr*	Monoplex -qPCR *InvA*	Multiplex -qPCR *ttr*	Multiplex -qPCR *InvA*
**Sensitivity**	Moderate (62.88%)	Very high (99.53%)	Very High (95.06%)	High (90.30%)	High (89.41%)
**Specificity**	Very high (99.99%)	Very High (95.46%)	High (90.31%)	Very high (99.30%)	Very high (98.00%)
**Estimated time to** **assay result (10** **samples)**	3 –7 days	1.5 days including overnight Selenite F broth enrichment	1.5 days including overnight Selenite F broth enrichment	0.5 day	0.5 day
**Person-labour time** **requirement**	Very high	High	High	high	high
**Cost estimate**	Low	High	High	Very high	Very high
**Specialist** **equipment cost**	Low: can be done in a standard microbiology laboratory	High: qPCR machine	High: qPCR machine	High: TAC-compatible qPCR machine Compatible centrifuge buckets TAC plate sealer	High: TAC-compatible qPCR machine Compatible centrifuge Buckets TAC plate sealer
**Follow-up antibiotic** **sensitivity**	Possible	Not possible	Not possible	Not possible	Not possible
**Serotyping**	Possible	Possible using serotype- specific primers	Possible using serotype- specific primers	Possible using serotype-specific primers	Possible using serotype-specific primers
**Efficiency in** **batching**	Low: working on more samples at once reduces efficiency	Very High: batched samples can be efficiently extracted and tested 96 samples and controls can be tested using 1 plate	Very High: batched samples can be efficiently extracted and tested 96 samples and controls can be tested using 1 plate	Very High: batched samples can be efficiently extracted and tested 8 samples and multiple pathogens can be tested using 1 plate	Very High: batched samples can be efficiently extracted and tested 8 samples and multiple pathogens can be tested using 1 plate

The *ttr* primer/ probe set used in the monoplex qPCR was previously validated for use in food samples and required validation in stool specimens. Our in-house developed *InvA* primer/ probe set also needed validation. Both assays demonstrated that they could detect all the different *Salmonella* strains, including *S.* Enteritidis, *S.* Typhimurium, and *S.* Typhi strains which are the commonly isolated strains in Malawi and SSA ^45^. Comparing the limits of detection of different *Salmonella* isolate conditions demonstrated that selenite pre-culture achieves a significantly lower limit of detection (1 CFU/ml) as opposed to direct isolates (10 CFU/ml) and *Salmonella*-spiked stool (10 CFU/ml). Selenite F broth is a selective broth that suppresses fecal coliforms and streptococci growth to optimise *Salmonella* growth ^46^. The LOD achieved after sub-culturing samples in Selenite enrichment broth agrees with results demonstrated by other studies, including a study done by Boer *et al.,* who showed that sub-culturing samples in Selenite F broth promotes the recovery of *Salmonella* in stool samples and improves sensitivity if samples are subsequently tested using molecular methods like PCR ^46, 47^.

Given the lack of a true gold standard diagnostic test, we took a model-based approach and used an LMM to estimate the specificities and sensitivities of the 5 *Salmonella* detection methods. Stool culture demonstrated the highest specificity but had the lowest sensitivity. All molecular assays; TAC- *ttr*, TAC- *InvA*, *ttr*, and *InvA*, demonstrated high specificity and sensitivity rates. Compared to the other methods, the monoplex based qPCR *ttr* achieved, in our opinion, the best sensitivity-specificity trade-off as it demonstrates near-perfect sensitivity (99.53%) and still achieves high specificity (95.43%). While monoplex *ttr* has the best overall performance, depending on the research context or clinical purpose, practitioners may still prefer tests with higher specificity such as stool culture, TAC- *ttr,* or TAC- *InvA*. All molecular test methods had significantly higher sensitivities than stool culture. High specificity and low sensitivity rates for culture have been widely reported ^18^. Such low sensitivity rates should be taken into consideration when evaluating diagnostic tests. It is clear that a reference test with poor sensitivity is not adequate to evaluate alternative test methods. In such a situation, alternative means of evaluating the assays should be used, such as the LMM that has been used here. LMMs, and their counterpart for cross-sectional data, latent class models (LCMs), have been used to evaluate diagnostic tests for various pathogens, including *Salmonella* ^48^.

PPV and NPV vary depending on the prevalence of the condition being tested in any particular population. Our samples were collected from a population that was considered healthy and asymptomatic at the time of recruitment. Using the model-estimated stationary probability of being infected, we estimated the Salmonella infection prevalence of 5.25% in this population. With this prevalence estimate, stool culture demonstrated a high PPV (99.73%) when compared to molecular tests that had high NPVs, with the highest NPV recorded by mono- *ttr* (99.67%). As a trade-off between PPV and NPV, in a population of asymptomatic children in urban Blantyre, TAC- *ttr* achieved high PPV (85.97%) and high NPV (90.33%). Whether a test with high PPV or NPV is to be preferred depends on the research and/or clinical context. When prevalence is low, a slight change in specificity will have significant effects on the PPV. Higher PPVs could be observed in a situation where prevalence is high such as when using a cohort of hospitalised diarrheal cases or during a diarrhoeal outbreak.

Molecular methods had higher sensitivity but lower specificity relative to stool culture. The loss in specificity is slight compared to the gain in sensitivity, and in the case of *Salmonella*, the public health cost of false-negative results could be higher if the infection becomes potentially life-threatening due to withholding or delay of treatment. With the high sensitivity, molecular methods were able to detect asymptomatic *Salmonella* events, critical for the research questions we hoped to pose in this cohort. All the events that were detected here were asymptomatic in healthy children, which are potentially very important in transmission or the development of immunity. The detection of low bacterial burden events could also be relevant in settings like Malawi where unprescribed over-the-counter antibiotic procurement and use is common. Studies that have reported on risk factors of having a culture-negative result have indicated that antibiotic usage before sample collection is the main risk factor. Using molecular techniques such as PCR could overcome this challenge because it detects bacterial DNA regardless of pathogen viability. This might increase the probability of identifying the infection and reduce sample processing time, leading to proper patient management and treatment if needed.

Our study has several limitations. One main limitation is the use of different sample types for the two qPCR platforms. The use of selenite sub-cultured stool samples in monoplex qPCR may have contributed to the superior performance when compared with the multiplex qPCR. We used neat stool samples for multiplex qPCR to comply with the manufacturer’s protocol. Other studies have, however, demonstrated that testing primer/ probe sets in the monoplex platform perform better than in the multiplex qPCR platform. Clinical samples used to test the performance of the test are a limitation, especially in determining the PPV and NPV. Clinical samples used in the study were collected from a cohort of children that were asymptomatic to *Salmonella* and remained healthy for most of the one-year study period. Using samples from participants with a clinical diagnosis of Salmonella or diarrhoea would have resulted in PPV and NPV estimates more directly relevant in a clinical setting. Another important limitation is the model-based nature of our approach. This was a necessity given the lack of a gold standard diagnostic test but does mean that our estimates depend on the validity of the model’s assumptions, in particular, i) the assumption that the latent infection state at a given time only depends on the immediate previous timepoint, the so-called Markov assumption, and ii) the conditional independence assumption of the basic LMM. While our modelling framework had been extended to relax the latter assumption, a better fit was achieved for the basic model.

## Conclusion

The data presented here demonstrate that the addition of selenite F broth pre-enrichment step increases *Salmonella* detection in stool samples and that *ttr* and *InvA* primer and probe sets used can detect different Salmonella strains. The ability of *ttr* to detect *Salmonella* with such high levels of specificity and sensitivity when tested using clinical samples collected from a mostly healthy cohort makes it a promising assay that could be used for research surveillance studies. The assays could be very useful in studying the transmission of *Salmonella* infections. This method may perform with different sensitivity and specificity in a chronic carriage, diarrhoeal or invasive *Salmonella* disease state, since the load and culturability of the pathogen within the stool may be different, and further validation studies would be needed

We established that selenite pre-culture increased diagnostic yield for molecular detection and identified *ttr* primers as molecular tools that could best help reveal the true extent of *Salmonella* exposure events within the gastrointestinal tract. This will allow us to understand their importance to diarrhoeal and invasive disease pathogenesis and epidemiology in the future.

## Data availability

### Underlying data

Figshare: Data and software code for Bayesian mixed latent Markov models for binary diagnostic data, https://doi.org/10.6084/m9.figshare.12911870.

1. gitMarcH-Bayesian-mixed-latent-Markov-models-for-binary-diagnostic-data.zip (software code for Latent Markov Model used in this study)

2. Data files used by the uploaded software code:
  - salexpoLIMSDataSetComplete.csv (Date of sample collection and follow-up visit number)
  - TACResults_4Mar TAC *ttr* TAC *InvA* Ct For Correlation.csv (Ct values for TAC_ *ttr* and TAC_ *InvA*)
  - *ttr* & *InvA* master file Ct for correlation.csv (Ct values for monoplex *TTR* and *InvA*)
  - *TtrInvA*Sensitivity20170724_corrected.csv (Combined binary results for stool culture, *ttr*, *InvA*, TAC_ *ttr,* and TAC_ *InvA* used to calculate sensitivity, specificity and correction of the test methods)

3. Raw data:
  - TAC Results_TAC- *ttr*_TAC- *InvA*_I_Ct ValuesTAC Results_TAC- *ttr*_TAC- *InvA*_IC_Ct-values.csv (raw Taqman array card assay results for test and control sample)
  - Salmonella_Detection_Stool_ *ttr*_ *InvA*_raw_data.xlsx (raw data for the monoplex qPCR assay. Includes results for test and control sample)

Data are available under the terms of the Creative Commons Attribution 4.0 International license (CC-BY 4.0).
