# Supplementary material for: Performance of molecular methods for the detection of Salmonella in human stool specimens
Source: Wellcome Open Res. 2021 May 4;5:237. Originally published 2020 Oct 9. [Version 2] doi: 10.12688/wellcomeopenres.16305.2 (PMC8108707; doi:10.12688/wellcomeopenres.16305.2)
Supplement: Peer review history

# Peer review: Reviewer response for version 2

**DOI:** 10.21956/wellcomeopenres.18556.r43663

**Published:** 2021-05-06

**Authors:** JY Zhang

**Affiliations:** 1 National Institute for Communicable Disease Control and Prevention, Beijing, China

The revised manuscript has improved a lot. Some details are not concise and accurate, but they provide new ideas for the evaluation of salmonella detection methods.

Is the rationale for developing the new method (or application) clearly explained?

Yes

Is the description of the method technically sound?

Yes

Are the conclusions about the method and its performance adequately supported by the findings presented in the article?

Partly

If any results are presented, are all the source data underlying the results available to ensure full reproducibility?

Yes

Are sufficient details provided to allow replication of the method development and its use by others?

Partly

Reviewer Expertise:

Molecular detection of intestinal pathogens.

I confirm that I have read this submission and believe that I have an appropriate level of expertise to confirm that it is of an acceptable scientific standard.

# Peer review: Reviewer response for version 2

**DOI:** 10.21956/wellcomeopenres.18556.r43662

**Published:** 2021-05-05

**Authors:** Qiuchun Li

**Affiliations:** 1 Key Laboratory of Prevention and Control of Biological Hazard Factors (Animal Origin) for Agri-food Safety and Quality, Ministry of Agriculture of China, Yangzhou University, Yangzhou, China

The manuscript has been revised seriously and is acceptable for indexing.

Is the rationale for developing the new method (or application) clearly explained?

Partly

Is the description of the method technically sound?

Partly

Are the conclusions about the method and its performance adequately supported by the findings presented in the article?

Partly

If any results are presented, are all the source data underlying the results available to ensure full reproducibility?

Partly

Are sufficient details provided to allow replication of the method development and its use by others?

Partly

Reviewer Expertise:

Prevalence of Salmonella in Foods and humans.

I confirm that I have read this submission and believe that I have an appropriate level of expertise to confirm that it is of an acceptable scientific standard.

# Peer review: Reviewer response for version 1

**DOI:** 10.21956/wellcomeopenres.17920.r41999

**Published:** 2021-01-27

**Authors:** Qiuchun Li

**Affiliations:** 1 Key Laboratory of Prevention and Control of Biological Hazard Factors (Animal Origin) for Agri-food Safety and Quality, Ministry of Agriculture of China, Yangzhou University, Yangzhou, China

This study used four different methods (monoplex- qPCR TTR, monoplex- qPCR invA, multiplex TAC-TTR, multiplex TAC InvA) in detecting *Salmonella* in stool samples with comparison to the traditional stool culture methods. The manuscript is not well-written and hard to follow. I have some major questions:

1. Application of these methods into 421 stool samples, 40, 29, 56, and 47 samples were detected positive using mono-TTR, mono-InvA, TAC-TTR, and TAC-InvA, respectively. While 23 samples were detected positive using the stool culture method. the study concluded the detection methods using TTR primers could be used for research surveillance studies, but didn’t confirm which method is reliable and applicable? And why?
2. When using the mon-TTR and mono-InvA, the genomic DNA used is from the selenite broth cultured samples, which is time-consuming for cultivation of the stool samples, so what is the advantage of these methods?
3. The specificity of the molecular methods used only 9 *Salmonella* serotypes, why not use some frequently reported human nontyphoidal *Salmonella* serotypes like *S.* London, *S.* Rissen, and *S.* Meleargidis, *S.* senftenberg, etc.? In addition, it is better to give the background of the prevalence of TTR and InvA in *Salmonella* serotypes based on genome sequencing analysis. The TAC is also used multiplex PCR methods, there is no detailed information on TAC in the manuscript.
4. The PPV and NPV have also been used to evaluate the molecular detection methods, under the condition of an estimated 5.25% of *Salmonella* in asymptomatic people, which method is better based on analysis of PPV and NPV?
5. It is necessary to revise the English grammar of the manuscript.

Is the rationale for developing the new method (or application) clearly explained?

Partly

Is the description of the method technically sound?

Partly

Are the conclusions about the method and its performance adequately supported by the findings presented in the article?

Partly

If any results are presented, are all the source data underlying the results available to ensure full reproducibility?

Partly

Are sufficient details provided to allow replication of the method development and its use by others?

Partly

Reviewer Expertise:

Prevalence of Salmonella in Foods and humans.

I confirm that I have read this submission and believe that I have an appropriate level of expertise to state that I do not consider it to be of an acceptable scientific standard, for reasons outlined above.

# Response

**Published:** 2021-04-12

**Authors:** Angeziwa Chirambo

**Affiliations:** Malawi Liverpool Wellcome TrusT Clinical Research Programme, Malawi

Reviewer Report 2

*27 Jan 2021 | for Version 1*

 Reviewer: Qiuchun Li

 Affiliation: Key Laboratory of Prevention and Control of Biological Hazard Factors (Animal Origin) for Agri-food Safety and Quality, Ministry of Agriculture of China, Yangzhou University, Yangzhou, China 

 Outcome: Not Approved

***Reviewer general comment:***

 This study used four different methods (monoplex- qPCR TTR, monoplex- qPCR invA, multiplex TAC-TTR, multiplex TAC InvA) in detecting *Salmonella* in stool samples with comparison to the traditional stool culture methods. The manuscript is not well-written and hard to follow. I have some major questions:

***Author's general response***

 More effort has been made to make the manuscript clear by carefully proof-reading of the manuscript and correcting any grammatical errors and have reviewed some of the scientific content as detailed in the below responses.

***Reviewer comment 1:***

**Application of these methods into 421 stool samples, 40, 29, 56, and 47 samples were detected positive using mono-TTR, mono-InvA, TAC-TTR, and TAC-InvA, respectively. While 23 samples were detected positive using the stool culture method. The study concluded the detection methods using TTR primers could be used for research surveillance studies but didn't confirm which method is reliable and applicable? And why?

***Author's response 1:***

 We thank the reviewer for highlighting that the main conclusion was not stated clearly enough. Mono-TTR is the method which, based on our data and analysis approach, achieves, in our opinion, the best trade-off between sensitivity and specificity. However, depending on context and purpose, other researchers and practitioners may prefer to prioritise sensitivity or specificity differently and therefore prefer one of the other methods we discussed.

 We have clarified this in the Results and Discussion sections. In the Results section, we now state:

*"While stool culture achieved the highest specificity and monoplex TTR the highest sensitivity, monoplex TTR achieved arguably the best trade-off between sensitivity-and specificity: very high sensitivity (99.53%) at a relatively small drop in specificity (95.46) compared to stool culture.”*

 In the Discussion section, we now clarify:

*"Compared to the other methods, the monoplex based qPCR TTR achieved, in our opinion, the best sensitivity-specificity trade-off as it demonstrates near-perfect sensitivity (99.53%) and still achieves high specificity (95.43%). While monoplex TTR has the best overall performance, depending on the research context or clinical purpose, practitioners in different contexts may still choose tests with higher specificity such as stool culture, TAC-TTR or TAC-InvA".*

 As per our response to reviewer 1, point 3, we also more clearly stated that our test performance characteristics have been derived using a statistical model. In Methods, we now state:

*"Another important limitation is the model-based nature of our approach. This was a necessity given the lack of a gold standard diagnostic test but does mean that our estimates depend on the validity of the model's assumptions, in particular i) the assumption that the latent infection state at a given time only depends on the immediate previous timepoint, the so-called Markov assumption, and ii) the conditional independence assumption of the basic LMM. While our modelling framework had been extended to relax the latter assumption, the better fit was achieved for the basic model."*

***Reviewer comment 2:***

 When using the mono-TTR and mono-InvA, the genomic DNA used is from the selenite broth cultured samples, which is time-consuming for cultivation of the stool samples, so what is the advantage of these methods?

***Author's response 2:***

 A table showing the characteristics of *Salmonella* stool culture, Monoplex -qPCR *ttr, *Monoplex -qPCR *InvA, *Multiplex -qPCR *ttr and *Multiplex -qPCR *InvA* has been added to summarise the pros and cons of the test methods (Table 3).

 A table has been added to summarise the characteristics of the different salmonella detection methods used in this study in order to highlight the strengths and weaknesses of the methods used.

***Reviewer comment 3:***

 The specificity of the molecular methods used only 9 *Salmonella* serotypes, why not use some frequently reported human nontyphoidal *Salmonella* serotypes like *S.* London, *S.* Rissen, and *S.* Meleargidis, *S.* senftenberg, etc.? In addition, it is better to give the background of the prevalence of TTR and InvA in *Salmonella* serotypes based on genome sequencing analysis.

***Author's response 3:***

 The following additions have been made to the Methods section subsection "Monoplex- qPCR ttr and InvA assay"

*“The use of ttr was based on* *(Malorny et al., 2004)* *. In this study, the specificity of the ttr gene was assessed using 110 Salmonella strains representing 31 serotypes, and it demonstrated 100% specificity. The InvA gene has been widely studied and used as a pan Salmonella marker. In* *(Malorny, Hoorfar, Bunge, & Helmuth, 2003)* *, the specificity of InvA gene was assessed using 242 Salmonella strains representing 43 serotypes. It also demonstrated 100% specificity. InvA has shown 100% specificity in several other studies.”*

 The following additions have been made to the Methods section, subsection "Specificity of ttr and InvA primer/probe set for Salmonella compared to other local pathogens"

*“To determine the prevalence of ttr and InvA in the genomes of Salmonella serotypes, NCBI nucleotide blast was conducted (10 Feb 2021). Ttr and InvA nucleotide sequences were used as the query sequence against Salmonella enterica subspecies enterica (taxid:59201) genomes with a maximum target sequence set at 1000. Both primer sequences demonstrated 100% identity for most of the commonly isolated Salmonella serotypes.”*

*“For the invtro work, All Salmonella isolates with known serotypes available in the MLW blood culture isolate archive were used.”*

 Regarding the reviewers comments on TAC methodology, more details on TAC has been added to the manuscript (Methods section, subsection "Detection of Salmonella using multiplex qPCR assay

*"* *The performance of  the TAC method has been previously described and has now been widely used. It is used to detect multiple enteric pathogens, including bacteria, viruses, protozoa, and helminths. Targets included on the TAC card for pan Salmonella detection are InvA and ttr. Phocine Herpesvirus (PHhv) and MS2 targets are included as internal positive controls*. *"*

**

***Reviewer comment 4:***

**The PPV and NPV have also been used to evaluate the molecular detection methods, under the condition of an estimated 5.25% of *Salmonella* in asymptomatic people, which method is better based on analysis of PPV and NPV?

***Author's response 4:***

 PPV and NPV results are stated in Table 3 and Figure 1B. However, we feel it is more useful to compare the different methods using sensitivity and specificity. These are direct characteristics of the diagnostic tests whereas PPV and NPV depend on the prevalence of the condition being assessed in the population that is being studied. As such, our results for PPV and NPV are applicable in the context of an asymptomatic (at recruitment) population of infants in urban Blantyre, Malawi and are not immediately generalisable to other contexts. This said, according to Table 3 and Figure 1B, stool culture achieves the best PPV and mono-TTR the best NPV (in both cases virtually 100%). Overall best trade-off is probably achieved by TAC-TTR achieving both high PPV and NPV– but again it depends on the purpose of the researcher / clinician whether to prioritise high PPV or high NPV or something in-between. We have clarified this in the manuscript. In Results we now state:

*"While stool culture has highest PPV (99.73%) and mono-TTR the largest NPV (99.67%), arguably the best PPV and NPV trade-off is achieved by TAC-TTR (both high PPV, 85.97%, and high NPV, 90.33%). We note that these PPV and NPV estimates are for an asymptomatic population of children in urban Blantyre, Malawi and are not directly generalisable to different contexts and populations where prevalence may be higher or lower."*

 And in the Discussion section we now state:

*"PPV and NPV vary depending on the prevalence of the condition being tested in any particular population. Our samples were collected from a population that was considered healthy and asymptomatic at the time of recruitment. Using the model-estimated stationary probability of being infected, we estimated the Salmonella infection prevalence of 5.25% in this population. With this prevalence estimate, stool culture demonstrated a high PPV (99.73%) when compared to molecular tests that had high NPVs, with the highest NPV recorded by mono-TTR (99.67%). As a trade-off between PPV and NPV, in a population of asymptomatic children in urban Blantyre, TAC-TTR achieved both high PPV (85.97%) and high NPV (90.33%). Whether a test with high PPV or NPV is to be preferred depends on the research and/or clinical context."*

***Reviewer comment:***It is necessary to revise the English grammar of the manuscript.

**Author's response:** We have done a careful proof-reading of the manuscript and corrected any grammatical errors.

- Is the rationale for developing the new method (or application) clearly explained?

***Reviewer comment:***Partly

***Author's response:***We trust that the amendments made now fully describe the rationale.

- Is the description of the method technically sound?

***Reviewer comment: ***Partly

***Author's response:***We trust that the amendments made now give a complete technical description.

- Are sufficient details provided to allow replication of the method development and its use by others?

***Reviewer comment:*** Partly

***Author's response:***We trust that the amendments made now mean this method could be fully replicated.

- If any results are presented, are all the source data underlying the results available to ensure full reproducibility?

***Reviewer comment:*** Partly

***Author's response:*** The reviewer makes no specific comment above relating to this. We believe that in keeping with Reviewer 1's opinion, all data are available to make the results reproducible.

- Are the conclusions about the method and its performance adequately supported by the findings presented in the article?

***Reviewer comment:***Partly

***Author's response:*** We trust that the amendments made above mean that the conclusions are fully supported by the findings.

**Competing Interests**

 No competing interests were disclosed.

**Reviewer Expertise**

 Prevalence of Salmonella in Foods and humans.

**I confirm that I have read this submission and believe that I have an appropriate level of expertise to state that I do not consider it to be of an acceptable scientific standard, for reasons outlined above.**

 Henrion, M. Y. R., Chirambo, A., Nyirenda, T.S., Gordon, M. (2018). Mixed latent Markov models for longitudinal multiple diagnostics data with an application to Salmonella in Malawi. *JSM Proceedings Vancouver, Canada,*.

 Malorny, B., Hoorfar, J., Bunge, C., & Helmuth, R. (2003). Multicenter validation of the analytical accuracy of Salmonella PCR: towards an international standard. *Appl Environ Microbiol, 69*(1), 290-296. doi:10.1128/aem.69.1.290-296.2003

 Malorny, B., Paccassoni, E., Fach, P., Bunge, C., Martin, A., & Helmuth, R. (2004). Diagnostic real-time PCR for detection of Salmonella in food. *Appl Environ Microbiol, 70*(12), 7046-7052. doi:10.1128/aem.70.12.7046-7052.2004

# Peer review: Reviewer response for version 1

**DOI:** 10.21956/wellcomeopenres.17920.r41992

**Published:** 2021-01-21

**Authors:** JY Zhang

**Affiliations:** 1 National Institute for Communicable Disease Control and Prevention, Beijing, China

The authors optimized an in-house monoplex real-time PCR for the detection of *Salmonella  ttr* and *invA* genes in stool, validated their specificity, and assessed their performance using clinical stool specimens collected from a cohort of 50 asymptomatic healthy children. The main problems:

1. Nucleic acid detection of enrichment culture can obtain the highest sensitivity, and the sensitivity of culture method is poor, which many studies have mentioned. The authors need to summarize the innovation points again to make it more attractive.
2. Unlike other studies, this study used continuously collected specimens from cohort. If the authors can analyze the queue data, it will be more meaningful.
3. When calculating the sensitivity and specificity of a method, it is necessary to determine whether the tested object is true positive or true negative. Whether the specimen is really positive can be determined by test. The author's conclusion was based on statistical analysis rather than experimental results, which I don't think is ideal. 

Minor: 

- Introduction: Gene names should be in italics and lowercase.
- Methods:
   
  - Salmonella stool culture: The volume of selenite F broth should be given. Did this method follow a literature or is it used in the authors' laboratory? Inoculating the centrifuged pellet onto XLD, I think there are too many bacteria, which may affect the subsequent selection of suspected colonies.
  - Table 1: The fluorescent groups and quenching groups of the probes needed to be given.
  - Statistical analysis: I don't understand the method used by the authors, so I don't comment on it. I suggest that the authors should explain the reliability of this method.
- Results and discussion. The analysis of cohort data is more meaningful. The reasons for the inconsistent results should be analyzed.

Is the rationale for developing the new method (or application) clearly explained?

Yes

Is the description of the method technically sound?

Yes

Are the conclusions about the method and its performance adequately supported by the findings presented in the article?

Partly

If any results are presented, are all the source data underlying the results available to ensure full reproducibility?

Yes

Are sufficient details provided to allow replication of the method development and its use by others?

Partly

Reviewer Expertise:

Molecular detection of intestinal pathogens. I'm not familiar with the statistical method mentioned by the author, so I suggest other reviewers review it again.

I confirm that I have read this submission and believe that I have an appropriate level of expertise to confirm that it is of an acceptable scientific standard, however I have significant reservations, as outlined above.

# Response

**Published:** 2021-04-12

**Authors:** Angeziwa Chirambo

**Affiliations:** Malawi Liverpool Wellcome TrusT Clinical Research Programme, Malawi

**Reviewer report 1**

**Reviewer:** JY Zhang

**Affiliation:** National Institute for Communicable Disease Control and Prevention, Beijing, China 

*21 Jan 2021 | for Version 1*

**Outcome:** Approved with reservations

***Reviewer 1 comment*:**

 The authors optimised an in-house monoplex real-time PCR for the detection of *Salmonella  ttr* and *invA* genes in stool validated their specificity and assessed their performance using clinical stool specimens collected from a cohort of 50 asymptomatic healthy children. The main problems:

***Reviewer comment 1*:**

 Nucleic acid detection of enrichment culture can obtain the highest sensitivity, and the sensitivity of the method is poor, which many studies have mentioned. The authors need to summarise the innovation points again to make it more attractive.

***Authors' response 1:***

 A table showing the characteristics of *Salmonella* stool culture, Monoplex -qPCR *ttr, *Monoplex -qPCR *InvA, *Multiplex -qPCR *ttr, and *Multiplex -qPCR *InvA* has been added to summarise the pros and cons of the test methods (Table 3).

 The following revision has been made to the discussion section of the manuscript

*“By contrast, the advantage of a monoplex test is that it is economical than the multiplex, faster than stool culture, even with the addition of the selenite enrichment step and allows for batch-processing of samples which increases the efficiency of the test method.”*

***Reviewer comment 2*:**

 Unlike other studies, this study used continuously collected specimens from cohorts. If the authors can analyse the queue data, it will be more meaningful.

***Authors' response 2:***

 We are not sure that we fully understand what the reviewer means by 'queue' data. We take it to mean that the reviewer has questions around our use of longitudinal data. The statistical model (Henrion, 2018) has been developed to take account of the longitudinal nature of the data, with repeated observations of the same individuals. The model explicitly models the progression over time of the underlying infection process. This is a statistically principled approach. The cohort data allows us to detect Salmonella exposure events as they happened – useful given that Salmonella prevalence can vary across the year.

 If we have misunderstood the reviewer's comment, we are happy to further elaborate on the nature of the data and our analysis approach.

***Reviewer comment 3*:**

 When calculating the sensitivity and specificity of a method, it is necessary to determine whether the tested object is true positive or true negative. Whether the specimen is really positive can be determined by test. The author's conclusion was based on statistical analysis rather than experimental results, which I don't think is ideal. 

***Authors' response 3:***

 We thank the reviewer for highlighting that we were not clear enough about how we estimated the test performances. The reviewer is right to point out that we used a statistical model for assessing the various diagnostic methods. If a true, perfect gold standard method existed, then it would, of course be preferable to compare the methods against this perfect diagnostic test. However, in our case the only available reference standard, culture, is known to have low sensitivity. For this reason, we decided to use the statistical model described in the manuscript and also, in more detail, in a separate publication (Henrion, 2018)

 The statistical model we used is of a particular type, specifically it is a latent Markov model (LMM), an accepted approach to model the underlying true infection state of individuals. The model uses this modelled latent infection state to estimate the sensitivity and specificity parameters that are most likely to have given rise to the observed data. As with any statistical model, there are a number of assumptions that have been made (in particular the LMM used in the present manuscript assumes i) that the infection state at a given timepoint only depends on the immediate prior timepoint and ii) that conditional on the latent state, the response probabilities of the different diagnostic tests are independent) and if these are not met, the results could be invalid.

 Despite the need to use a statistical rather than an experimental approach, we have done some experimental validation. using spiked stool samples to assess detection limits for the molecular methods. We could not, however, use such spiked samples to calculate sensitivities and specificities: such artificial samples do not reflect true, biological samples and could lead to estimates for sensitivity and specificity that are biased to incorrectly high values. For this reason, we preferred to use real world data and a statistical approach to best estimate the test characteristics. Please note that this is a well-recognised and validated approach in this situation, and similar models to ours have been used by other authors to evaluate novel diagnostic tests (Koukpunari A, 2013)

 We have made the following revisions to the manuscript to incorporate and clarify the above points:

- In the Discussion section where we discuss the specificities and sensitivities that we estimated, we now clarify this is a model-based approach:

*"Given the lack of a true gold standard diagnostic test, we took a model-based approach and used an LMM to estimate the specificities and sensitivities of the 5 Salmonella detection methods."*

  

- In the Discussion section, we now list the model-based approach as another limitation, given that our results depend on the validity of the model assumptions. We now state:

*"Another important limitation is the model-based nature of our approach. This was a necessity given the lack of a gold standard diagnostic test but does mean that our estimates depend on the validity of the model's assumptions, in particular) the assumption that the latent infection state at a given time only depends on the immediate previous timepoint, the so-called Markov assumption, and ii) the conditional independence assumption of the basic LMM. While our modelling framework had been extended to relax the latter assumption, the better fit was achieved for the basic model.”*

 Minor: 

***Reviewer's comment*:** Introduction: Gene names should be in italics and lowercase.

***Authors' response:*** Gene names have been changed to italics and lowercase

***Reviewer's comment:*** Methods:

 Salmonella stool culture: The volume of selenite F broth should be given. Did this method follow a literature or is it used in the authors' laboratory? Inoculating the centrifuged pellet onto XLD, I think there are too many bacteria, which may affect the subsequent selection of suspected colonies.

***Authors' response:***

 The following changes have been made in the methods section under the subsection "Salmonella stool culture"

1. “ *10 mls of selenite F broth was used. THis is a method that has been developed in our own laboratory.”*
2. *"1* *m l loop was used to subculture Salmonella from the pellet in order to achieve single colonies.”  *

***Reviewer's comment:***

 Table 1: The fluorescent groups and quenching groups of the probes needed to be given.

***Author's response:***

 The fluorescent and quenching groups have been added to the sequences for probes

***Reviewer's comment:***

 Statistical analysis: I don't understand the method used by the authors, so I don't comment on it. I suggest that the authors should explain the reliability of this method.

***Author's response:***

 We thank the reviewer for highlighting the difficulty to understand the model approached used. We will attempt to clarify the main ideas behind the modelling framework in this response. The necessity for a model-based approach is due to the fact that there is no good reference standard diagnostic method available. While our modelling framework allows for different complexity of data, the basic LMM used in this manuscript consists of two parts: i) a latent or unobserved process model for the underlying infection status, ii) a measurement model for the observed test results from the 5 diagnostic methods used. The process model estimates where a given study participant is infected or not and how the transitions between infected and non-infected progress over time. The measurement model estimates the probabilities of recording a positive, respectively a negative result, given whether or not the study participant is infected. These probabilities from the measurement model are the test sensitivities (probability of recording a positive result when infected) and specificities (probability of recording a negative result when not infected).

 LMMs, and the related latent class analysis models for cross-sectional data, have been used extensively for evaluating novel diagnostic methods (e.g. (Koukpunari A, 2013; van Smeden, Naaktgeboren, Reitsma, Moons, & de Groot, 2014) With the caveat that results are only as valid as the model's assumptions, such models provide a reliable evaluation of diagnostic methods when there is no true gold standard. We have previously evaluated, using simulated data where the true infection state is known, the reliability of the LMM modelling framework used in the present manuscript (Henrion, 2018).

 While we already refer to a separate paper describing the details of the model  (Henrion, 2018), we have added the following clarification in the Methods section:

*"LMMs consist of a process model for a latent condition (in our case the unobserved true infection status) evolving over time and a measurement model for the observed outputs (in our case the results from the 5 diagnostic test methods) conditional on the latent state".*

 Results and discussion. 

***Reviewer's comment:***

 The analysis of cohort data is more meaningful. The reasons for the inconsistent results should be analysed.

***Author's response:***

 This point is not **very clear to us. We **would be happy to address this comment if the reviewer would shed more light.

- Is the rationale for developing the new method (or application) clearly explained?

Yes

- Is the description of the method technically sound?

Yes

- Are sufficient details provided to allow replication of the method development and its use by others?

***Reviewer's comment:*** Partly

***Author's response:*** Additional technical information has been added as requested

- If any results are presented, are all the source data underlying the results available to ensure full reproducibility?

Yes

- Are the conclusions about the method and its performance adequately supported by the findings presented in the article?

***Reviewer's comment:*** Partly

***Author's response:***

 We trust that the clarifications requested and made in the manuscript now adequately support the conclusions

**Competing Interests**

 No competing interests were disclosed.

**Reviewer Expertise**

 Molecular detection of intestinal pathogens. I'm not familiar with the statistical method mentioned by the author, so I suggest other reviewers review it again.

**I confirm that I have read this submission and believe that I have an appropriate level of expertise to confirm that it is of an acceptable scientific standard, however I have significant reservations, as outlined above.**

 Henrion, M. Y. R., Chirambo, A., Nyirenda, T.S., Gordon, M. (2018). Mixed latent Markov models for longitudinal multiple diagnostics data with an application to salmonella in Malawi. *JSM Proceedings Vancouver, Canada,*.

 Koukpunari A, D., C. A., …, et al., . (2013). A latent Markov modelling approach to the evaluation of circulating cathodic antigen strips for schistosomiasis diagnosis pre- and post-praziquantel treatment in Uganda. *PLOS Computational Biology,*.

 van Smeden, M., Naaktgeboren, C. A., Reitsma, J. B., Moons, K. G., & de Groot, J. A. (2014). Latent class models in diagnostic studies when there is no reference standard--a systematic review. *Am J Epidemiol, 179*(4), 423-431. doi:10.1093/aje/kwt286
